# Environmental effects on mineral supplement intake in grazing Nellore heifers under tropical conditions

**DOI:** 10.1007/s11250-026-04945-7

**Published:** 2026-02-24

**Authors:** Bruna Cardoso Braga, Marinaldo Divino Ribeiro, Raphael dos Santos Gomes, Luiz Gustavo Barbosa, Severino Delmar Junqueira Villela, Terry E. Engle, Fernando de Paula Leonel

**Affiliations:** 1 Department of Animal Science, Federal University of Góias, Goiânia, GO 74691-835 Brazil; 2 Federal Institute of Education, Science and Technology of Rondonia, Porto Velho, RO 76803-260 Brazil; 3 Department of Animal Science, Federal University of Jequitinhonha and Mucuri Valleys, Diamantina, MG 39100-000 Brazil; 4 Department of Animal Sciences, Colorado State University, Fort Collins, CO 80523 USA; 5 Department of Animal Science, Federal University of São João del-Rei, São João del- Rei, MG 36301-360 Brazil

**Keywords:** Grazing behavior, Climate variability, Feed intake, Smart feeders, Animal welfare, Tropical systems, Bos indicus, Precision livestock monitoring

## Abstract

Environmental factors can influence nutrient intake in grazing cattle, yet few studies have evaluated how such conditions affect mineral supplement intake. This study assessed the effects of ambient temperature, rainfall, and seasonal variation on mineral supplement intake in Nellore heifers under tropical grazing conditions. Thirty-six heifers (initial age: 9 ± 1 months.; final age: 28 months; initial and final average body weights: 170 and 386 kg, respectively) were managed in a continuous grazing system on a 24 ha pasture of *Brachiaria brizantha* cv. MG4. Individual supplement intake was recorded via electronic smart feeders (Intergado^®^), and environmental data were collected hourly from 09:00 to 18:00 h during a 21-month experimental period. Days were categorized based on the thermal comfort zone (TCZ) for zebu cattle (10 °C to 27 °C) as: within TCZ, below 10 °C (TL10), or above 27 °C (TH27). Supplement intake was evaluated in relation to hours spent outside the TCZ, rainfall occurrence, and season (dry or wet). Average intake on days within the TCZ did not differ from TL10 or TH27 (*P* = 0.07). However, as the duration of thermal stress increased, supplement intake and feeder visitation rates declined (*P* < 0.001). Heifers exposed to more than three hours below or two hours above the TCZ showed the lowest intakes. Rainfall and season had no effects on mineral intake. Prolonged exposure to environmental temperatures outside the TCZ negatively affects mineral supplement intake, highlighting the importance of considering climatic stressors in pasture-based nutrition programs.

## Introduction

Thermal stress, a common challenge in tropical regions, can reduce feed intake and impair performance in grazing cattle (Souza et al. 2007; Azevedo et al. 2005; Façanha et al. 2010). In Brazil, seasonal variation is primarily defined by rainfall, with a wet season ranging from October to March and a dry season spanning from April to September. In tropical grazing systems, cattle are frequently exposed to intense solar radiation and thermal extremes, which may lead to heat stress and modifications in feeding and grazing behavior (Mitlöhner et al. 2002; Manteca and Smith 1994).

Nellore cattle, widely used in tropical production systems have morphological and physiological adaptations—such as efficient heat dissipation capacity, increased sweat gland density, and pigmented skin—that enhance tolerance to high temperatures (De Melo Costa et al. 2017; Hooper et al. 2018; Cunningham and Syrstad 1987). The thermal comfort zone (TCZ) for zebu cattle ranges from 10 to 27 °C (Baêta and Souza 1997; Santos et al. 2005; Furtado et al. 2012), and deviations from this range trigger thermoregulatory responses that may modify feeding patterns (Pereira 2005).

While the effects of environmental stress on dry matter intake are well known (McDowell 1985; Mitlöhner et al. 2002), few studies have addressed its impact on mineral supplement intake, despite its crucial role in supporting growth, reproduction, and immune function (Suttle 2010; Underwood and Suttle 2000; McDowell et al., 1996; Schatz et al. 2023). Moreover, rainfall and seasonal conditions may further influence grazing time and feeding access (Wyffels et al. 2020; DelCurto and Olson 2010; Walburger et al. 2009).

Thus, the objective of this study was to evaluate the influence of ambient temperature, rainfall, and seasonal variation on mineral supplement intake by Nellore heifers in a tropical grazing system, using individual real-time intake monitoring through smart feeders.

## Material and methods

All animal procedures were approved by the Animal Use Ethics Committee (CEUA; protocol number 048/24) and conducted in accordance with national and institutional guidelines.

The study was conducted in a region classified as Cwb (subtropical highland climate) according to the Köppen-Geiger system, characterized by mild temperatures and a well-defined dry winter and wet summer. During the experimental period, daily temperatures typically ranged from approximately 10 to 27 °C, consistent with the thermal conditions of tropical highland environments. The supplementation station used (smart feeders) had the following coordinates: 20º59’49.80”S; 44º23’52.41”W.

The data collection period (supplement intake and environmental variables) began in September 2020 and ended in May 2022, totaling 21 months. All information on temperature and rainfall was extracted from a meteorological station that is part of the network of the National Institute of Meteorology (INMET) of Brazil, located five km from the experimental area.

Data on the number of visits to the smart feeders and supplement intake were collected from 36 contemporary Nellore heifers. The experiment began when the heifers were 9 ± 1 months old with an average body weight of 170 kg. At the end of the experiment the heifers were 28 months old and had an average body weight of 386 kg. The database consisted of a total of 20,808 data points for supplement intake. The heifers remained in a continuous grazing system in an area of 24 hectares covered with *Brachiaria brizantha* cv. MG4. All the heifers received the supplement shown in Table 1.

**Table 1:** Ingredients inclusion and chemical composition of supplement

Ingredients	g/kg		Nutrients	g/kg
Corn ground	200		Calcium	77
Soybean meal	70		Phosphorus	38
Urea	120		Sodium	114
Limestone	90.1		Sulfur	5
Dicalcium phosphate	200		Magnesium	3
Sodium chloride	300		Cobalt	6
Elemental Sulfur	5.1		Copper	400
Magnesium oxide	4.8		Iron	500
Cobalt sulfate	0.030		Iodine	20
Copper sulfate	1.600		Manganese	900
Iron sulfate	1.786		Selenium	3
Calcium Iodate	0.032		Zinc	1200
Manganese sulfate	3.214		EM (Mcal/kg)	0.75
Sodium Selenite	0.007		CP	343
Zinc sulfate	3.429		NNP eq CP	338
Total	1000			

ME: Metabolizable Energy; CP: crude protein; NNP eq CP: Non-Protein Nitrogen equivalent to Crude Protein

The supplement was offered *ad libitum* to heifers, and individual access to smart feeders (Intergado^®^) was controlled by electronic ear tags. The smart feeders were equipped with load cells, antennas for recognition and release access for each animal, and data collectors. Data were sent via Wi-Fi to a central processor that released the output of the animals’ individual supplement intake information. Climatic data were collected hourly throughout the experimental period, from 09:00 h to18:00 h. This time of day was used to collect climate data, as it is the period in which animals visit the supplementation station most frequently.

The thermal comfort zone (TCZ) considered in this study was in the range of 10 to 27 °C, which is the thermal comfort zone for zebu cattle (Baêta and Souza 1997; Santos et al. 2005; Furtado et al. 2012). Days in which temperatures from 9 a.m. to 6 p.m. that remained within the thermal comfort zone for cattle (> 10 °C and < 27 °C) were classified as days within TCZ. However, when the temperature was below 10 °C or above 27 °C for at least one hour of the day, between 9 a.m. and 6 p.m., the day was classified as TL10 or TH27, respectively. This allowed for examining the effect of average temperature outside the TCZ as well as the effect of the number of hours below (TL10) or above (TH27) the TCZ on supplement intake by heifers. For clarity, the classification of days relative to the TCZ is summarized in Table 2.

**Table 2:** Classification of days according to temperature relative to the thermal comfort zone (TCZ)

Category	Definition	Temperature condition	Possible range (hours/day)
TCZ	Days when the temperature remains entirely within the thermal comfort zone	10 to 27 °C	0 h outside TCZ
TL10	Days with at least one hour below the TCZ	< 10 °C	1 to 6 h below TCZ
TH27	Days with at least one hour above the TCZ	> 27 °C	1 to 6 h above TCZ

During the experimental period, the animals were outside the TCZ, from 0900 h to 1800 h, for a maximum of 6 h per day. To evaluate the effect of the intensity of animal exposure to temperatures below and above the TCZ, days were classified as: 0 h (days within the TCZ); 1, 2, 3, 4, 5 and 6 h (hours of exposure to temperatures below the TCZ), and 1, 2, 3, 4, 5 and 6 h (hours of exposure to temperatures above the TCZ). To evaluate the frequency of visits to the supplementation station, days with intake equal to zero were considered as “absence at the supplementation station,” and any intake different from zero was considered as “presence at the supplementation station.” Therefore, the frequency of visits to the supplementation station was calculated as a percentage of days classified as presence at the supplementation station (DPSS) in relation to the total number of days in experiment (TNDE): DPSS/TNDE x 100.

Furthermore, the effects of dry and wet seasons, as well as the occurrence of rainfall, on supplement intake were evaluated. The dry season was considered from May to October and the wet season was considered from November to April.

Data on mineral supplement intake were analyzed according to a completely randomized design, where each heifer was considered an experimental unit. The following statistical model was used:

$$\:{y}_{ij}=\mu\:+{\tau\:}_{i}+{\epsilon\:}_{ij}$$

where:$\:{y}_{ij}$= observed value of supplement intake (g/day) of the *j*-th heifer under the *i*-th treatment;$\:\mu\:$= overall mean; $\:{\tau\:}_{i}$= fixed effect of the *i*-th treatment (i.e., temperature class, number of hours outside the thermal comfort zone, rainfall occurrence, or season); $\:{\epsilon\:}_{ij}$= random error, assumed to be normally and independently distributed with mean zero and variance $\:{\sigma\:}^{2}$.

The model was adjusted separately for each factor (temperature condition, season, and rainfall), and mean comparisons were performed at a 5% probability level using the R software (R Core Team, 2021).

To evaluate the percentage of visits to the supplementation station, the Kruskal-Wallis test was used at a 5% probability level in the R software (R Core Team, 2021).

## Results

**Fig. 1:**
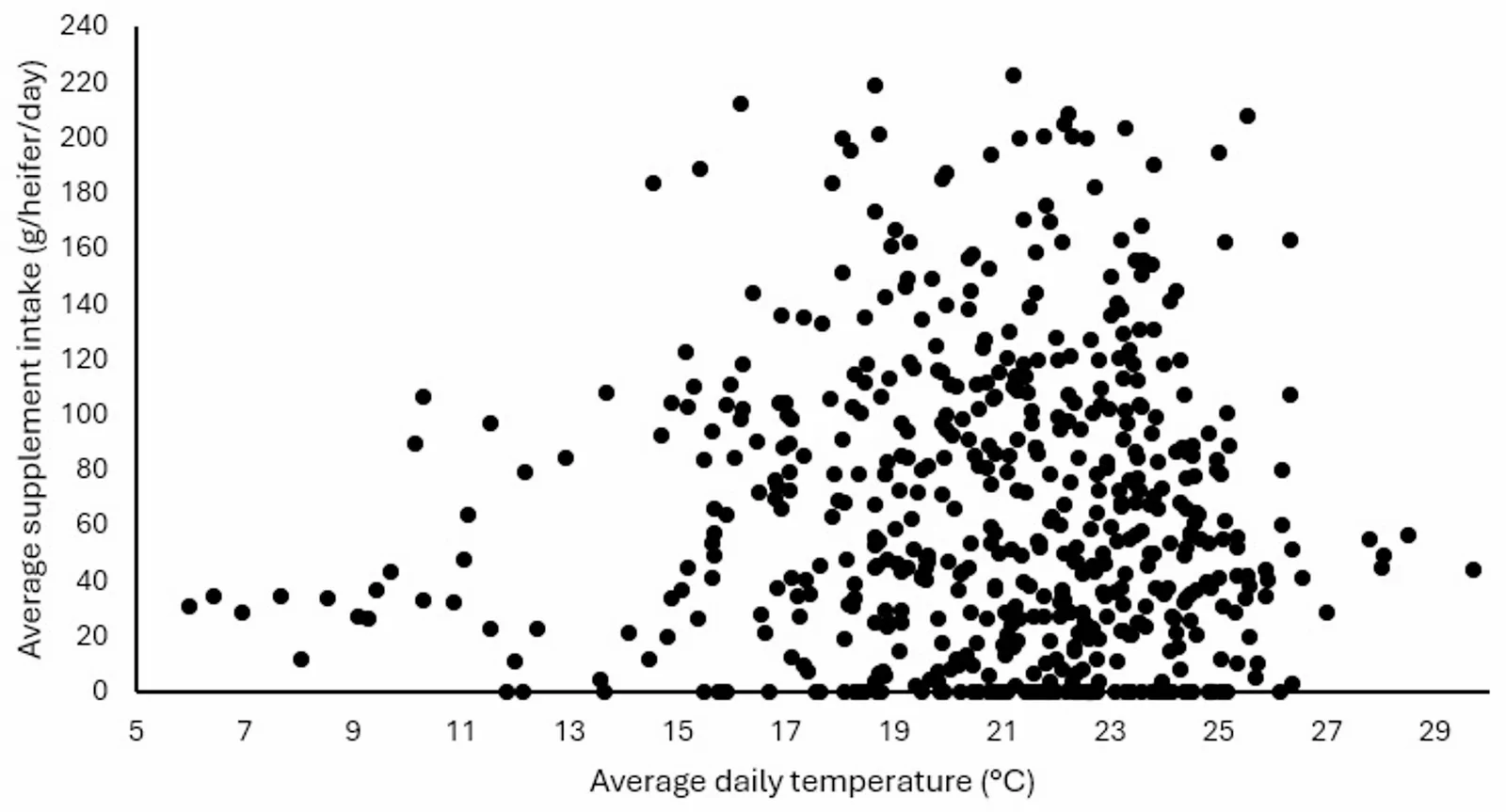
Average supplement intake (g/heifer/day) as a function of average daily temperature (°C)

The dispersion of average supplement intake as a function of average daily temperature is shown in Fig. 1. The average supplement intake on days with average temperatures within the TCZ did not differ from days in which the average temperature reached values lower than 10 °C (TL10) or higher than 27 °C (TH27; *P* = 0.0643; Table 3).

**Table 3:** Average, minimum and maximum temperatures of the days and average supplement intake (g/heifer/day) followed by the standard error in parentheses, depending on the different temperature ranges

Temperature values	TL10	CTZ	TH27
Average (°C)	14.8	19.4	22.8
Minimum (°C)	6	10.9	11.1
Maximum (°C)	25	26.9	29.7
Supplement Intake	68.08 (6.14)	74.07 (3.31)	62.10 (2.87)

TL10: days when temperatures below 10 °C were recorded; TCZ: days with temperatures between 10 °C and 27 °C; TH27: days with temperatures higher than 27 °C

Heifers ingested more (*P* < 0.001) supplement on days with up to two hours of temperatures in TL10 than on days with temperatures in the TCZ. On days with three hours in TL10, supplement intake did not differ significantly (*P* > 0.05) when compared to days within the TCZ. On days with more than three hours in TL10, heifer supplement intake was lower (*P* < 0.001) than in the TCZ, with a progressive decrease up to six hours in TL10 (Table 4).

**Table 4:** Average daily supplement intake (g/heifer/day, ADSI) and frequency of visits to the supplementation station, according to the number of hours per day of temperature below the TCZ

Hours	ADSI	SE	Total days^1^	Days with visits^2^	% visits^3^		
6 H	19.44 ^d^	19.44	2	1		50.00 ^d^	
5 H	42.11 ^c^	16.33	12	7		58.33 ^d^	
4 H	58.75 ^c^	9.78	25	17		68.00 ^cd^	
3 H	74.80 ^b^	11.00	14	10		85.71 ^abc^	
2 H	94.04 ^a^	16.72	7	7		100.00 ^ab^	
1 H	113.92 ^a^	11.37	7	7		100.00 ^ab^	
TCZ	74.01 ^b^	3.31	182	171		93.92 ^a^	

* Within column, values followed by different letters are significantly different (*P* < 0.05). ** SE: standard error

^1^ Number of total days within each classification; ^2^ Number of days the heifers visited the supplementation station; ^3^ Percentage of visits depending on the number of total days in each classification calculated by the Kruskal-Wallis test

Heifers visited the supplement feeders more frequently (*P* < 0.001) on days with temperatures within the TCZ and on days with up to two hours in TL10. On days with three or more hours of temperatures in TL10, there was a gradual decrease (*P* < 0.001) in the number of visits to the smart feeder (Table 4).

With up to two hours a day of temperatures in TH27, there were no differences (*P* < 0.001) in supplement intake compared to days within the TCZ. However, supplement intake decreased (*P* < 0.001) on days in which heifers were exposed to more than three hours per day in TH27, with a progressive decrease until six hours in TH27. The lowest supplement intakes were on the days when the heifers were exposed to 6 h in TL10 and TH27 (Table 5). When exposed to temperatures above the TCZ for one hour or more, heifers reduced (*P* < 0.001) the frequency of visits to the supplement feeders (Table 5).

**Table 5:** Average daily supplement intake (g/heifer/day, ADSI) and frequency of visits to the supplementation station, according to the number of hours per day of temperature above the TCZ

Hours	ADSI	SE	Total days^1^	Days with visits^2^	% visits^3^
TCZ	74.01 ^b^	3.31	182	171	93.92 ^a^
1 H	68.94 ^b^	11.37	32	25	78.12 ^bc^
2 H	82.05 ^b^	7.65	59	53	89.83 ^ab^
3 H	63.12 ^c^	6.01	64	53	82.81 ^bc^
4 H	53.04 ^c^	5.16	89	64	71.91 ^cd^
5 H	58.49 ^c^	5.64	66	57	86.36 ^ab^
6 H	46.64 ^c^	10.47	27	21	77.78 ^bc^

** Within column, values followed by different letters are significantly different (*P* < 0.05). ** SE: standard error

^1^ Number of total days within each classification; ^2^ Number of days the animals visited the supplementation station; ^3^ Percentage of visits depending on the number of total days in each classification calculated by the Kruskal-Wallis test

The average annual rainfall recorded in the rainy seasons during the experimental period was: 1682 mm from 01/11/20 to 30/04/21 and 2626 mm from 01/11/21 to 30/04/22. The presence or absence of rainfall did not affect (*P* = 0.7099) supplement intake by heifers. Also, this supplement intake was not different (*P* = 0.2135) depending on the seasons, dry or rainy (Table 6).

**Table 6:** Average daily supplement intake (g/heifer/day, ADSI), followed by the standard error in parentheses, depending on the presence or absence of rainfall and the season (dry/wet)

Variables	Rainfall			Season	
	No	Yes		Dry	Wet
Minimum rainfall (mm/event)	0	0.2		-	-
Maximum rainfall (mm/event)	0	62.8		-	-
Temperature, average (°C)	-	-		18.82	22.3
Temperature, minimum (°C)	-	-		6.00	16.8
Temperature, maximum (°C)	-	-		29.7	26.6
ADSI	67.16 (2.39)	65.43 (4.28)		69.70 (3.07)	64.42 (2.85)
Days number^1^	413	165		246	332

^1^number of days with or without rainfall and number of days in the dry or wet season

## Discussion

Regardless of the variables under study, supplement intake by heifers was lower than expected. This lower supplement intake may be attributed to design constraints of the smart feeders. To ensure accurate individual recording, the device restricts access to one animal at a time. Considering the social behavior of cattle, this restriction may result in lower supplement intake compared to systems where all animals have simultaneous access to feeders (Stricklin and Kautz-Scanavy 1984).

### Physiological arguments

The thermal comfort zone (TCZ) is limited by the lower and upper critical temperatures. Within TCZ, animals have less physiological expenditure to maintain homeostasis and therefore can divert excess energy to growth. When the temperature is below the lower critical limit, beef cattle increase heat production which reduces that amount of energy directed to growth (Young and Christopherson 1974; Webster 1970, 1971). Conversely, if cattle are exposed to a temperature above the upper critical limit, the animal will reduce heat production, increase heat dissipation mechanism (sweating, panting, etc.) therefore reducing the amount of energy that can be directed to growth (Pereira 2005).

The average temperatures recorded during the experimental period were mild, and on most days the records were within TCZ, with few records below TL10 or above TH27. Therefore, there was no thermal challenge for Nellore breed heifers, which, due to the evolutionary process, adapt well to the climate of tropical regions (Cunningham and Syrstad 1987). These animals have morphological adaptations such as a greater number of sweat glands, greater surface area which facilitates heat dissipation (Zanette and Kruger 2011). Also, they have a skin highly pigmented, covered with white, short, dense fur. The white fur coat reflects infrared radiation, which is calorific, favoring thermal balance, as it reduces heat gained from the environment. In addition, it has physiological evolutions that enable less heat production and greater efficiency in thermal dissipation (Cunningham and Syrstad 1987).

There are no reports in literature describing the impact of temperature variations on supplement intake in Nellore beef heifers. Wyffels et al. (2020) studied the behavior of supplement intake in a grazing system. However, their study was with cows receiving a supplement with greater intake potential and was restricted to the winter period, which is more severe than the conditions imposed in our study. However, the current study revealed a reduction in supplement intake by Nellore heifers exposed to more than four hours a day at temperatures lower than 10 °C or more than three hours a day at temperatures higher than 27 °C.

### Behavioral arguments

The lower supplement intake on days with a greater number of hours outside the TCZ may not be a direct consequence of physiological mechanisms to compensate for heat stress. This lower intake may be a consequence of a behavioral strategy to gain or lose heat. In other words, on days with a greater number of hours below TL10 or above TH27, heifers visit the supplementation station less. However, this lower number of visits may have different causes: (1) days with a greater number of hours below TL10, the animals may reduce visits to the feeders to conserve heat and remain clustered in the field, thus reducing heat loss to the environment and, (2) days with temperatures above TH27, the animals may have remained longer in shaded areas and, as a result, the frequency of visits to the supplementation station decreases. Thus, the lower intake of supplements on days with a greater number of hours outside the thermal comfort zone can be attributed to the lower frequency of visits to the supplementation station.

Variations on the intake of the supplements with low fermentative potential (mineral supplements) in function of changes in environmental temperature should not be explained based in classic studies about the effect of thermal stress on dry matter intake. This is because, in the total diet, heat of fermentation and metabolic heat can impact heat production in cattle (McDowell 1985; Mitlöhner et al. 2002). However, in low intake supplements with a predominantly mineral composition, physiological mechanism does not act in this way, as these supplements are ingested in small daily quantities and have a low capacity to supply rumen fermentable substrates.

There are few studies that address the influence of climatic variables such as temperature and rainfall on the intake of mineral supplements. However, a parallel can be made with studies on the reduction of grazing time by animals subjected to high temperatures (Manteca and Smith 1994). The lack of influence of the season (dry or wet) on supplement intake can be explained by the adaptive characteristics of the zebu breed. These characteristics are responsible for excellent adaptation in a tropical environment. The lack of an effect of the rainfall on supplement intake is probably due to the dynamics of rainfall, which is frequently at night, when the animals do not visit the supplementation station. When rainfall occurs during the daylight, it often happens for a short period of time, so the animals still have.

From a practical standpoint, the patterns observed in this study provide relevant guidance for cattle producers operating in tropical grazing systems. The reduction in mineral supplement intake during periods of extended exposure outside the TCZ suggests that environmental conditions can jeopardize the consistency of mineral consumption, particularly when animals reduce feeder visitation due to heat or cold avoidance strategies. To mitigate these effects, producers may benefit from positioning supplementation stations closer to shaded areas or along natural congregation points, especially during seasons or hours of the day when heat load is greater. Placing feeders near windbreaks or sheltered zones may help maintain normal visitation behavior in colder periods. Additionally, ensuring multiple access points or increasing feeder availability can alleviate the social and behavioral restrictions. Strategic timing of supplement placement and monitoring weather forecasts can further support consistent intake during anticipated thermal challenges. Collectively, these practices may help stabilize mineral intake, safeguard nutritional adequacy, and improve overall herd performance under fluctuating climatic conditions, as well as increase the time available to visit the supplementation stations.

## Conclusion

Exposure to temperatures outside the TCZ influenced the mineral supplement intake of Nellore heifers. Prolonged daily exposure to temperatures below 10 °C or above 27 °C reduced supplement intake, mainly due to decreased visitation to the supplementation station. In contrast, exposure of up to two hours below the TCZ increased intake, indicating that mild cold conditions may stimulate supplement consumption.

Rainfall occurrence and seasonal variation (dry versus wet season) did not affect mineral supplement intake. These findings suggest that short-duration rainfall events and the strong environmental adaptability of zebu cattle minimize seasonal impacts on supplement use.

Overall, the results demonstrate that climatic conditions can influence the consistency of mineral supplement intake in tropical grazing systems. Understanding these dynamics supports the development of more precise nutritional management strategies, particularly in designing supplementation access, positioning of feeders relative to shade, and adjusting management practices during periods with greater thermal variation.

## Data Availability

The data that support the findings of this study are available from the corresponding author upon reasonable request.
